# ILs-3, 6 and 11 increase, but ILs-10 and 24 decrease stemness of human prostate cancer cells *in vitro*

**DOI:** 10.18632/oncotarget.5883

**Published:** 2015-10-21

**Authors:** Dandan Yu, Yali Zhong, Xiaoran Li, Yaqing Li, Xiaoli Li, Jing Cao, Huijie Fan, Yuan Yuan, Zhenyu Ji, Baoping Qiao, Jian-Guo Wen, Mingzhi Zhang, Gunnar Kvalheim, Jahn M. Nesland, Zhenhe Suo

**Affiliations:** ^1^ Department of Oncology, The First Affiliated Hospital of Zhengzhou University, Zhengzhou University, Henan, China; ^2^ Departments of Pathology, The Third Affiliated Hospital of Zhengzhou University, Zhengzhou, Henan, China; ^3^ Department of Pathology, Capital Medical University, Beijing, China; ^4^ Department of Oncology, Henan Academy of Medical & Pharmaceutical Sciences, Zhengzhou University, Zhengzhou, China; ^5^ Department of Urodynamic Center and Urology, The First Affiliated Hospital of Zhengzhou University, Zhengzhou University, Henan, China; ^6^ Department of Cell Therapy, Cancer Institute, Radium Hospital, Oslo University Hospital, Oslo, Norway; ^7^ Department of Pathology, The Norwegian Radium Hospital, Oslo University Hospital, Oslo, Norway; ^8^ Department of Pathology, Institute of Clinical Medicine, Faculty of Medicine, University of Oslo, Oslo, Norway

**Keywords:** interleukin, cytokines, cancer stem cells, chemotherapy, CD44 and ABCG2

## Abstract

Cancer stem cells (CSCs) are associated with cancer recurrence and metastasis. Prostate cancer cells often metastasize to the bone with a complex microenvironment of cytokines favoring cell survival. In this study, the cell stemness influence of a group of interleukins including IL-3, 6, 10, 11 and 24 on human prostate cancer cell lines LNCaP and PC-3 was explored *in vitro*. Sulforhodamine B(SRB) and 5-ethynyl-2′-deoxyuridine (EdU) assays were applied to examine the effect on cell proliferation, and wound healing and transwell assays were used for migration and invasion studies, in addition to colony formation, Western blotting and flowcytometry for the expression of stemness factors and chemotherapy sensitivity. We observed that ILs-3, 6 and 11 stimulated while ILs-10 and 24 inhibited the growth, invasion and migration of both cell lines. Interestingly, ILs-3, 6 and 11 significantly promoted colony formation and increased the expression of SOX2, CD44 and ABCG2 in both prostate cancer cell lines. However, ILs-10 and 24 showed the opposite effect on the expression of these factors. In line with the above findings, treatment with either IL-3 or IL-6 or IL-11 decreased the chemosensitivity to docetaxel while treatment with either IL-10 or IL-24 increased the sensitivity of docetaxel chemotherapy. In conclusion, our results suggest that ILs-3, 6 and 11 function as tumor promoters while ILs-10 and 24 function as tumor suppressors in the prostate cancer cell lines PC-3 and LNCaP *in vitro*, and such differences may attribute to their different effect on the stemness of PCa cells.

## INTRODUCTION

Prostate cancer(PCa) is the second leading cause of cancer death among men in the European and American, followed by lung cancer [1], and the morbidity and mortality are rapidly increasing in China [2, 3]. Prostate specific antigen (PSA) is a specific marker for PCa, especially in the early stage. The androgen deprivation and radical prostectomy has been the gold standard therapy. Often PCa patients have a progression without clinical symptoms and the disease is detected in advanced stages.

The standard treatment for patients with primary hormone-dependent prostate cancer is endocrine therapy, and the treatment originally inhibits tumor growth. However, nearly all patients who received this treatment will ultimately relapse and develop bone metastases [4]. Recently, increasing evidence indicates that there are cancer stem cells (CSCs) survived in circulating system or bone marrow of prostate cancer patients, which attribute to therapy-resistant and relapse [5–7]. The most notable features of these multipotent and self-renewing cells are their high survival capability and high resistance to chemotherapy.

CSCs are driving force for the onset, development and progression of various cancers. Recent studies point to the possibility that a number of cytokines in the tumor microenviroment [8, 9] influence the stemness of cancer cells and CSC properties, and the growth and metastasis of tumors [10–12].

It is believed that conventional treatment options eradicate the bulk of more differentiated tumor cell clones. However, CSCs survive most of the classical chemotherapy regimens and contribute to the tumor progression and recurrence [13–15].

Interleukins (ILs) are a subgroup of cytokines being primarily reported in leukocytes. IL-3 is produced in activated T cells and mast cells, and influences the production, differentiation and function of granulocytes and macrophages [16, 17]. It has been reported that IL-3 receptor(IL-3R) overexpression on leukaemia stem cell populations is a common occurrence, and therapeutic IL-3-IL-3R interference option already shown promising [18]. And importantly, the IL-3R(CD123) had been considered as a biomarker of leukemia stem cells [19, 20], although contradictory results exist in the literature that IL-3 has not direct relationship with the hematopoietic stem cells (HSCs) [21], or breast cancer [22].

IL-6, also called B-cell stimulatory factor-2 and interferon beta-2, is involved in different biological functions, including B lymph cell differentiation and myeloma and plasmacytoma growth [23, 24]. Recently, it was observed that IL-6 is secreted from tumor-associated macrophages (TAMs) in hepatocellular carcinoma (HCC) and involved in the HCC tumorigenesis and also the CSC expansion [25]. Similar findings are also reported in human colon cancer [26] and malignant transformation of rat mesenchymal stem cells (MSCs) [27].

IL-10, another immunomodulatory cytokine, had been used as vehicle of the neural stem/progenitor cells (NSPCs) into the central nervous system (CNS) to replace damaged cells and cure inflammation [28]. IL-10 is implicated in enhancing the ability of MSCs in anti-inflammatory application [29] and hematopoietic stem cell transplantation (HSCT) in treatment of the inflammatory bowel disease (IBD) [30]. Other studies have shown that IL-10 may inhibit the growth and differentiation of neural stem cells (NSCs) in normal adult brain [31], and help the MSCs to inhibit the mature of dendritic cells (DCs) by JAK1 and STAT3 signaling pathway [32].

IL-11 is also a secreted cytokine and involves in megakaryocytopoiesis, platelet production, osteoclast activation and inhibition of epithelial cell proliferation and apoptosis [33]. IL-11 is active in peripheral blood stem cell(PBSC) mobilisation [35]. Clinically, it has been shown that IL-11 can decrease the occurrence of the development of graft versus host disease (GVHD), which is induced by allogeneic hematopoietic stem cell transplantation (allo-HSCT) [34].

Increasing evidence indicates that IL-24 may suppress the growth of cancer stem cells. Further studies revealed that IL-24 effectively inhibited proliferation and angiogenesis of cancer cells, even reduce the percentage of breast cancer-initiating/stem cells [36] and mesenchymal stem cells (MSCs) [37] *in vivo*.

We have previously reported that granulocyte-macrophage colony stimulating factor (GM-CSF) and colony stimulating factor(CSF) stimulate the stemness of PCa cells [38]. In our present study, we have examined the effect of different interleukins (IL-3,6,10,11 and 24) on the cell growth, migration, invasion, apoptosis, colony formation capability and chemotherapy resistance of the androgen-dependent LNCaP and the androgen-independent cell line PC-3 PCa cell lines, aiming to explore whether these cytokines could influence the cell stemness of the PCa cells *in vitro*.

## RESULTS

### The effect on cell growth

**Figure 1:**
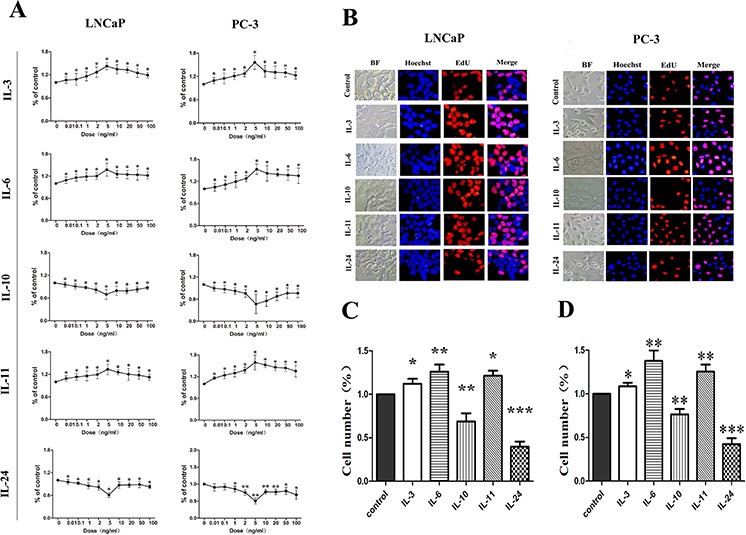
Growth influence of ILs on LNCaP and PC-3 cells **A.** shows growth curves of the dose dependent effect of the interleukins. There is no linear dose-dependent effect. Representative fluorescence microscopic images of the EdU and Hoechest 33342 staining are shown in **B.** for LNCaP and PC-3 cells, respectively. Both cells are stained with EdU (red) for DNA synthesis and Hoechest 33342(blue) for nuclear staining. **C.** and **D.** show histograms of the living cell numbers after treatments of the interleukins for 48 hrs for LNCaP and PC-3 cells, respectively. Data are presented as mean ± SD of three separate experiments. * means *p* < 0.05, ** means *p* < 0.01 and *** means *p* < 0.001, in comparison to the control groups, respectively.

In order to study the proliferation effect of the ILs on LNCaP and PC-3 cells, dose-dependent growth curves were made. As shown in Figure 1A, dose-dependent growth curves are not always lineage for all of these ILs. However, it is apparently from this figure that 5 ng/ml treatment is rather representative for all the ILs, and 5 ng/ml was therefore chosen to use for the following experiments. Representative images of fluorescence microscopy using the EdU incorporation and Hoechest 33342 for the cells treated for 24 hrs are shown in Figure 1B. Histograms of the fluorescence microscopy results are shown in Figure 1C and 1D for LNCaP and PC-3 cells, respectively. It was found that 24 hrs treatment with IL-3, IL-6 and IL-11 in 5 ng/ml significantly increased the cell numbers in both cells lines, with *p*-values of 0.02, 0.037 and 0.032 in LNCaP cells, and *p*-values of 0.043, 0.029 and 0.029 in PC-3 cells, respectively. However, 24 hrs treatment of IL-10 and IL-24 in 5 ng/ml significantly decreased the number of cells in both cell lines, with *p*-values of 0.023 and 0.018 in LNCaP cells, and *p*-values of 0.027and 0.029 in PC-3 cells, respectively. These results indicate that IL-3, IL-6 and IL-11 significantly stimulate the cell growth, while IL-10 and IL24 significantly inhibit the cell growth in both cell lines.

### The effect on cell mobility

**Figure 2:**
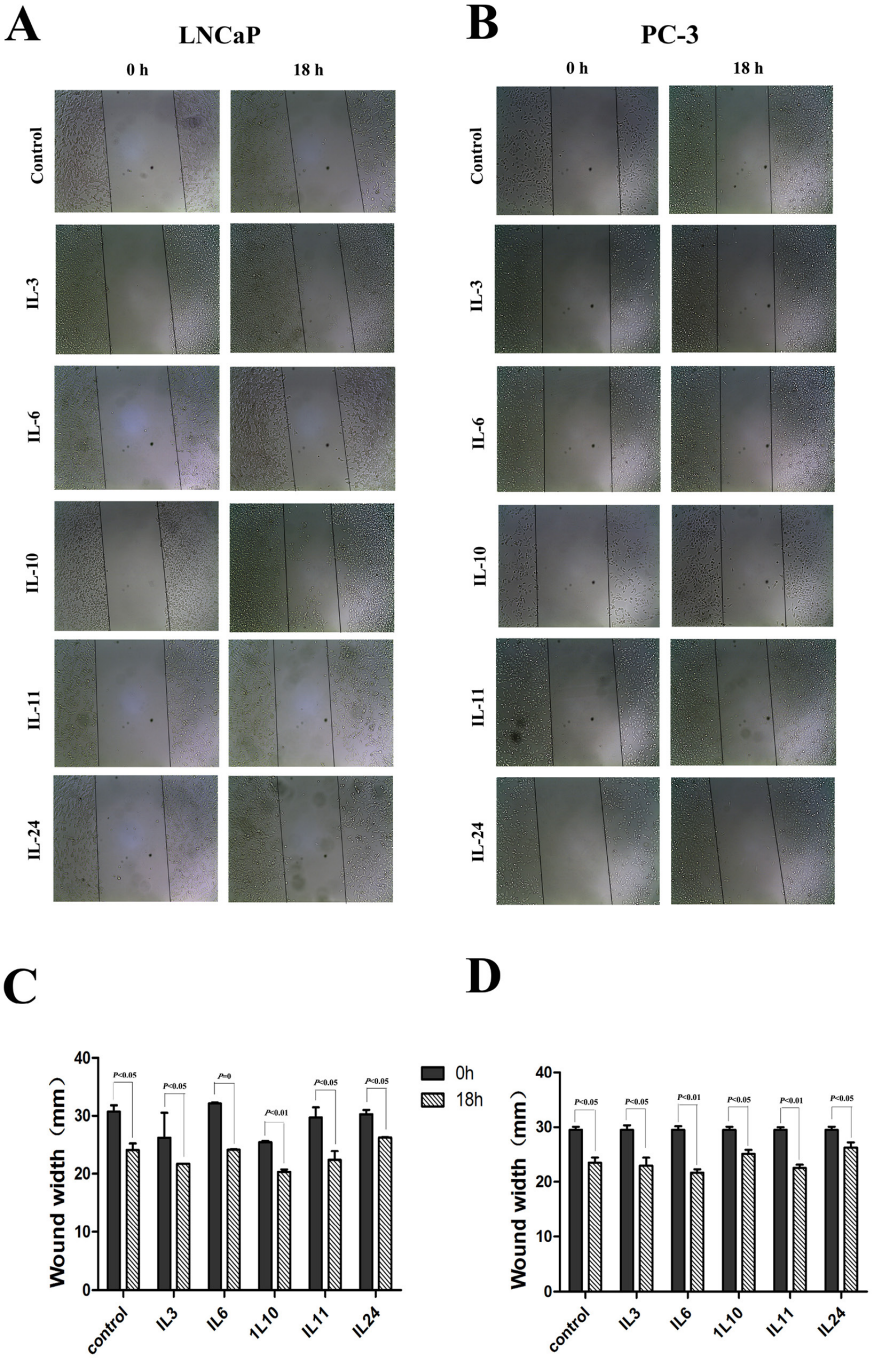
Results of wound healing assay **A.** and **C.** show representative images and histograms of the effect of different interleukins on LNCaP cell line, respectively. **B.** and **D.** show representative images and histograms of the effect of different interleukins on PC-3 cell line, respectively. Data are presented as mean ± SD of three separate experiments, *n* = 3. * means *p* < 0.05, ** means *p* < 0.01, and *** means *p* < 0.001, in comparison to the control groups, respectively.

The motility of human PCa cells lines LNCaP and PC-3 cells were examined by wound healing assay when treated with different ILs. Confluent monolayers of cells were scratched to be wounded and cultured for 18 hrs (Figure 2). Compared with the control cells, treatment with IL-3, IL-6 and IL-11 demonstrated significantly higher mobility in both cell lines, and the rates of wound healing increased 10.2%(*p* = 0.046), 21.1%(*p* = 0.004) and 11.9% (*p* = 0.047) in LNCaP cells and 13.6%(*p* = 0.049) 30.4%(*p* = 0.045) and 16.1%(*p* = 0.040) in PC-3 cells, respectively. But treatment with IL-10 and IL-24 showed an inhibition effect on the wound healing in comparison to the control cells, and the rates of wound healing decreased with 20.8%(*p* = 0.008) and 39.3%(*p* = 0.031) in LNCaP cells and 26.2%(*p* < 0.001) and 48.5%(*p* = 0.002) in PC-3 cells, respectively.

### Migration and invasion effect

**Figure 3:**
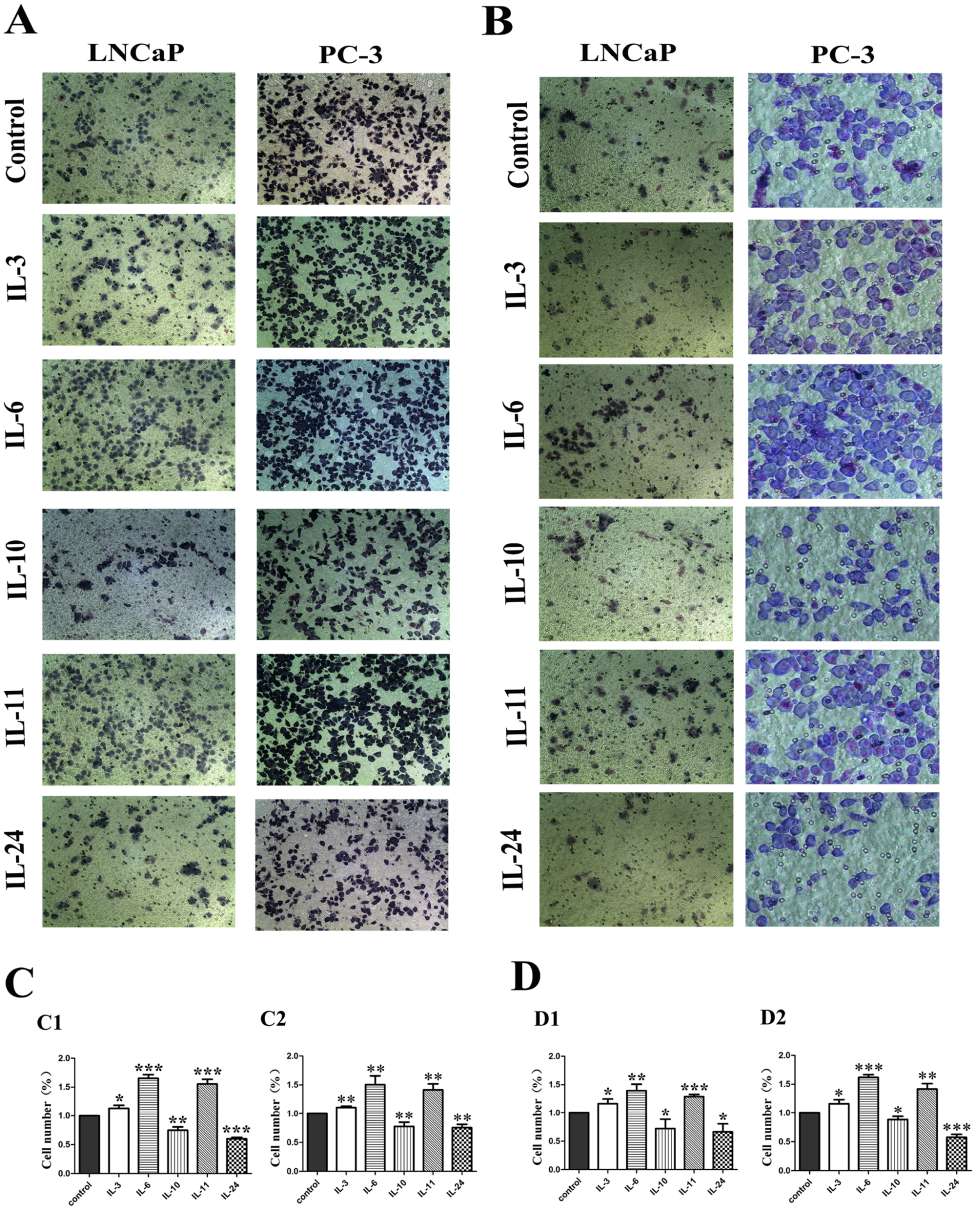
Migratory and invasion influence of ILs on LNCaP and PC-3 cells **A.** shows representative photographs of the cells migrated through the polycarbonate membrane stained by Gimsa. **B.** shows representative photographs of the invasive cells. **C.** shows histograms of the migration assay results and **D.** shows histograms of invasion assay results for both cell lines, respectively. While IL-3, IL-6 and IL-11 stimulate the migration and invasion of both cell lines, IL-10 and IL-24 significantly inhibit the migration and invasion of the cells as shown in C and D. All data represent means from three independent experiments. * means *p* < 0.05, ** means *p* < 0.01, and *** means *p* < 0.001.

A transwell chamber system was employed to measure the migration and invasion effect of different ILs on LNCaP and PC-3 cells. In general, migration and invasion ability of both cell lines was increased when treated with IL-3, IL-6 and IL-11, but decreased when treated with IL-10 and IL-24 (Figure 3A and 3B). When cell migratory ability was examined with the non-treated cells as controls in LNCaP cells, 24 hrs of IL-3, IL-6 and IL-11 treatment significantly increased the number of cells migrated through the membrane, with increased rates of 13.2% (*p* = 0.014), 65.3%(*p* = 0.014) and 55.4%(*p* < 0.001), respectively. However, 24 hrs of IL-10 and IL-24 treatment significantly decreased the number of cells migrated through the membrane, and the migration rates declined 25.3% and 40.0% with *p* = 0.002 and *p* < 0.001, respectively. The migratory effect on PC-3 cells was similar. Compared to the non-treated cells, 24 hrs of IL-3, IL-6 and IL-11 treatment significantly increased the number of cells migrated through the membrane with increased rates of 10.7% (*p* = 0.002), 50.5% (*p* = 0.004) and 41.2%(*p* = 0.002), respectively, while 24 hrs treatment of IL-10 and IL-24 significantly decreased the number of cells migrated through the membrane with decreased rates of 22.4% (*p* = 0.007) and 24.7% (*p* = 0.002), respectively(Figure 3C).

For cell invasion examination where the membrane was coated with 60 μL of matrigel, 24 hrs of IL-3, IL-6 and IL-11 treatment significantly increased the number of invasive cells. Compared with the control cells, the invasion rate increased 16.6% (*p* = 0.026), 39.5% (*p* = 0.004) and 28.9% (*p* < 0.001) in the IL-3, IL-6 and IL-11 treated LNCaP groups, and 16.3% (*p* = 0.017), 61.2% (*p* < 0.001) and 41.7% (*p* = 0.002) in the IL-3, IL-6 and IL-11 treated PC-3 groups, respectively. While 24 hrs of IL-10 and IL-24 treatment significantly decreased the number of cells penetrated through the membrane in both cell lines. Comparatively, the decreased invasion rates were 27.7% (*p* = 0.044) and 33.6% (*p* = 0.015) in the IL-10 and IL-24 treated LNCaP groups, and 27.7% (*p* = 0.023) and 42.3% (*p* < 0.001) in the IL-10 and IL-24 treated PC-3 groups, respectively (Figure 3D).

### The effect on chemotherapy resistance

The apoptotic effect of the ILs was firstly examined by flow cytometry.

**Figure 4:**
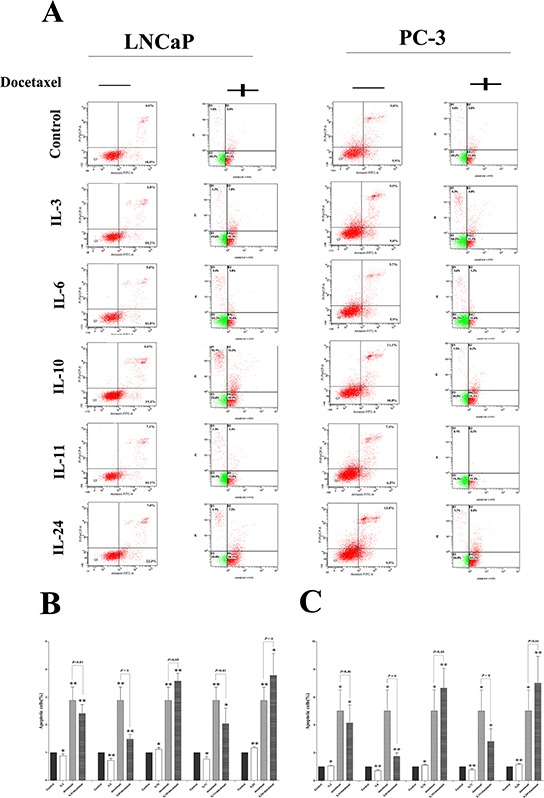
Docetaxel treatment sensitivity influence **A.** shows representative flowcytometry figures for LNCaP and PC-3 cell lines treated with different ILs with and without docetaxel application. Corresponding histogram results are shown in **B.** and **C.** for LNCaP and PC-3 cells, respectively. While docetaxel significantly induces apoptosis in both cell lines, application of either IL-3, or IL-6 or IL-11 slightly inhibits the apoptosis in the both cell lines. On the contrary, application of either IL-10 or IL-24 slightly increases the apoptosis already induced by the docetaxel. * means *p* < 0.05, ** means *p* < 0.01, and *** means *p* < 0.001, in comparison to the control groups, respectively.

Compared with the control cells, significantly lower numbers of apoptotic cells were seen in the cells treated with IL-3, IL-6 and IL-11 for 24 hrs, with *p*-values of 0.049, 0.003 and 0.011 in LNCaP cells, and *p*-values of 0.012, 0.001 and 0.002 in PC-3 cells, respectively. But treatment with IL-10 and IL-24 significantly increased the number of apoptotic cells, with *p*-values of 0.045 and 0.001 in LNCaP cells, and *p*-values of 0.040 and 0.009 in PC-3 cells, respectively (Figure 4).

The apoptotic effect of docetaxel on these cells was further examined. After optimization of the dose, 10nmol/L concentration of docetaxel was applied in this study. As shown in Figure 4, 24 hrs of docetaxel treatment alone significantly increased the number of apoptotic cells in these cell lines, with a *p*-value of 0.002 in LNCaP cells, and a *p*-value of 0.010 in PC-3 cells.

Then we asked whether the application of such ILs could influence the apoptosis influenced by docetaxel in these cells by joint application of the ILs and docetaxel. It was discovered that combination of docetaxel application with either IL-3 or IL-6 or IL-11 significantly reduced the numbers of apoptotic cells than those with docetaxel application alone, with *p*-values of 0.001, 0.007 and 0.017 in LNCaP cells, and *p*-values of 0.014, 0.002 and 0.026 in PC-3 cells, respectively. Also, the combination of docetaxel and either IL10 or IL-24 could significantly increase the number of apoptotic cells, with *p*-values of 0.001 and 0.024 in LNCaP cells, and *p*-values of 0.007 and 0.005 in PC-3 cells, respectively, indicating a chemo-sensitizing role of IL-10 and IL-24 in docetaxel treatment in prostate cancer cells.

### The effect on SOX2 expression

**Figure 5:**
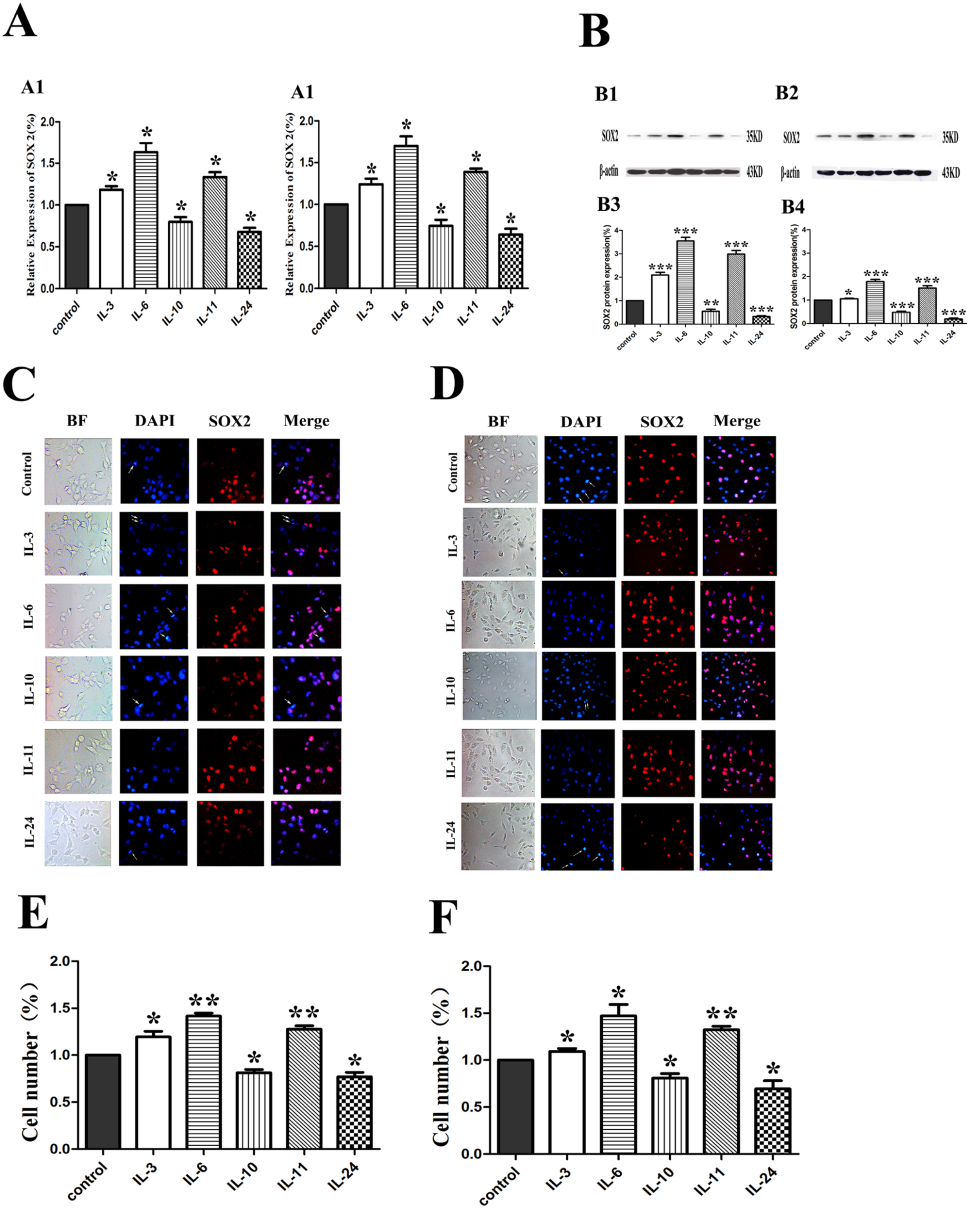
Influence of ILs on the RNA and protein expression of SOX2 in LNCaP and PC-3 cells by RT-PCR, Western blotting and immunofluorescence **A.** shows the relative gene expression of SOX2 figures for LNCaP and PC-3, respectively. **B.** shows the expression of SOX2 in LNCaP and PC-3 cells by Western blotting in protein level (upper) and the corresponding histograms (lower) in both cells. **C.** and **D.** show representative images of immunofluorescence microscopy of SOX2 in both cell lines. **E.** and **F.** show corresponding histograms of the SOX2 immunofluorescence microscopy for LNCaP and PC-3 cell lines, respectively. Cells are stained with DAPI to visualize the nuclei (blue). SOX2 (red) is localized in the nuclei. BF stands for bright field. All photographs were originally taken at 200 × .* means *p* < 0.05, and ** means *p* < 0.01.

To explore how ILs influenced the expression of SOX2, the expression of mRNA level of SOX2 was examines by RT-PCR(Figure 5A). Compared with the control groups, the expression of SOX2 mRNA was increased when treated with IL-3, IL-6 and IL-11 in both cells, while the expression of SOX2 mRNA when treated with IL-10 and IL-24, was decreased. All the mRNA level changes were consistent with the SOX2 protein expression alterations revealed with Western blotting as shown in Figure 5B. To further evaluate the expression of SOX2 in both cells, especially the cellular localization of SOX2, immunofluorescence microscopy of SOX2 was performed (Figure 5C–5F). All positive staining of the SOX2 in these cells was confined in the nuclei in both cell lines. Quantification of the positive cell numbers revealed similar results as demonstrated by RT-PCR (Figure 5A) and Western blotting (Figure 5B).

### Clonogenicity effect

**Figure 6:**
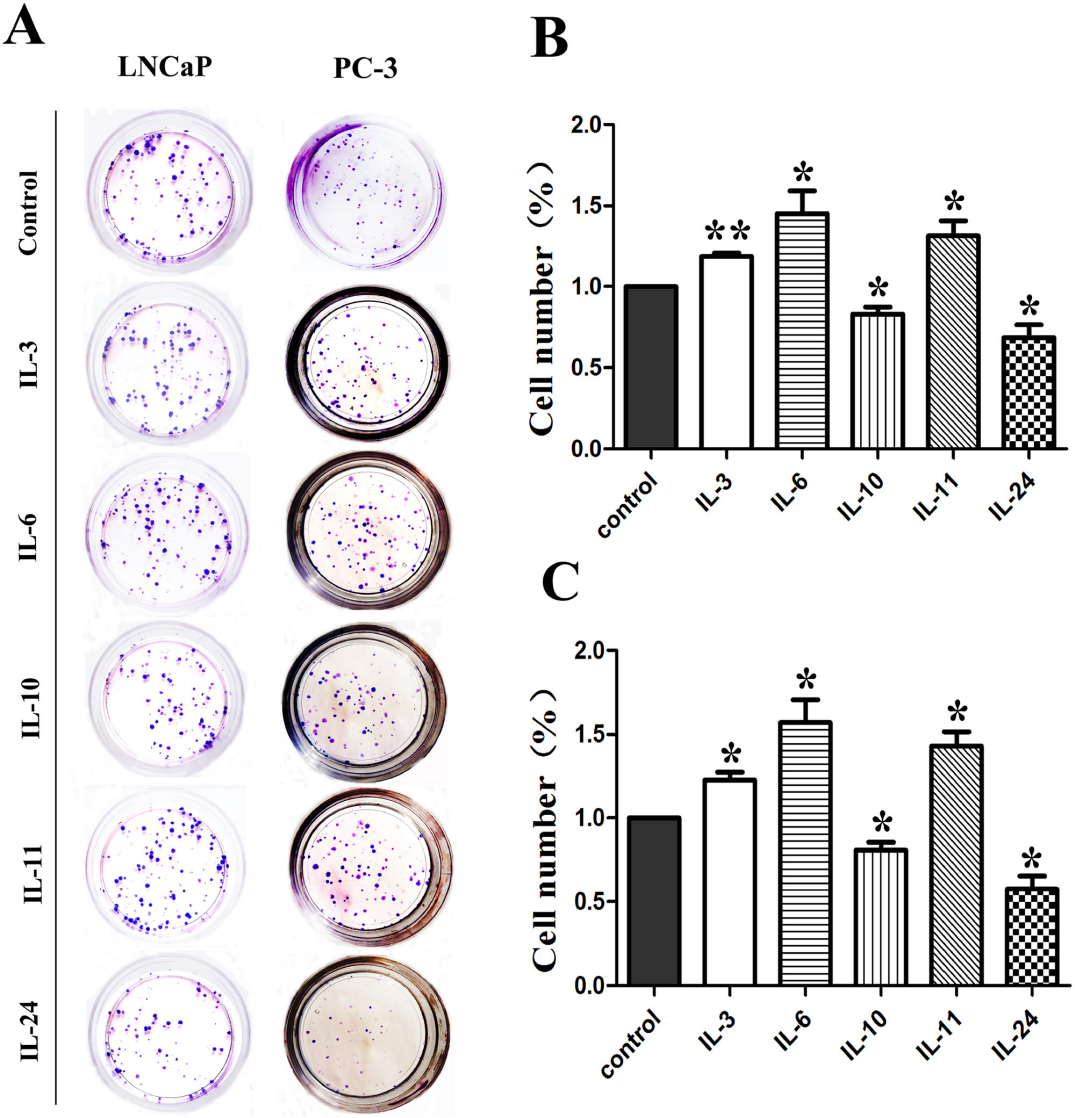
Results of colony formation assay Representative colony formation assay plates are shown in **A.** for both cell lines. Corresponding histograms for the effect of interleukins on LNCaP and PC-3 are shown in **B.** and **C.** respectively. * means *p* < 0.05 and ** means *p* < 0.01.

The colony formation assay was carried out to examine the clonogenicity effect of these cytokines. Representative photos of the colony formation assay for both cell lines are shown on Figure 6A, and corresponding histograms of the results are shown on Figure 6B for LNCaP cells, and Figure 6C for PC-3 cells. Compared to the control cells, treatment of IL-3, IL-6 and IL-11 increased the number of clones by 18.6% (*p* = 0.005), 45.3% (*p* = 0.045) and 31.3% (*p* = 0.042) in LNCaP cells as shown on Figure 6B, and by 22.6% (*p* = 0.021), 57.0% (*p* = 0.027) and 43.3% (*p* = 0.001) in PC-3 cells as shown on Figure 6C. However, treatment with IL10 and IL24 resulted in significantly fewer clones by 16.9% (*p* = 0.032) and 31.4% (*p* = 0.030) as shown on Figure 6B for LNCaP cells and 19.0% (*p* = 0.027) and 42.4% (*p* = 0.002) as shown on Figure 6C for PC-3 cells, respectively.

### The effect on the expression of CD44 and ABCG2

**Figure 7:**
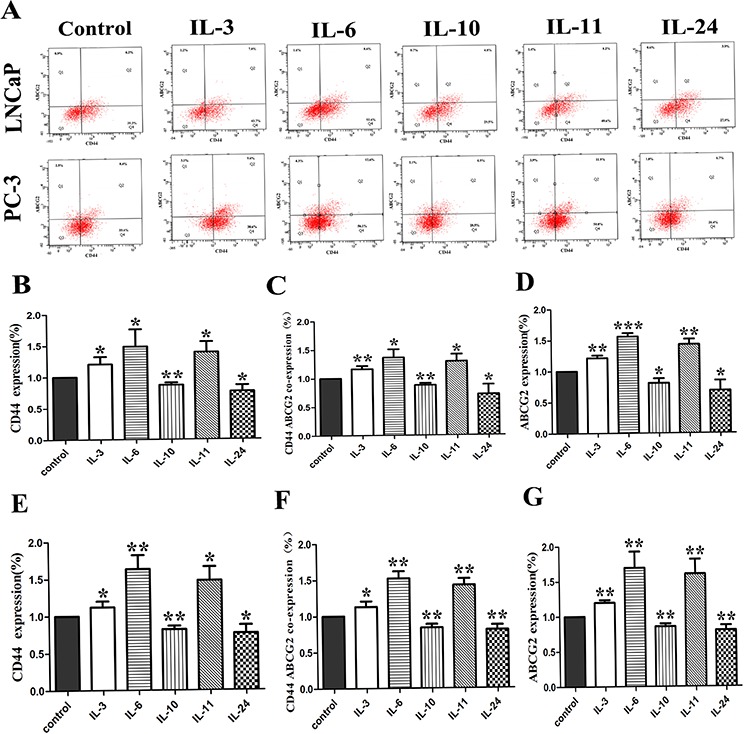
The effect of ILs on the expression of CD44 and ABCG2 **A.** shows representative flowcytometry of CD44 and ABCG2 in both cell lines. **B, C and D.** show corresponding histograms of CD44 alone, CD44 and ABCG2, and ABCG2 alone for LNCaP cells, respectively. **E, F** and **G.** show corresponding histograms of CD44 alone, CD44 and ABCG2, and ABCG2 alone for PC-3 cell lines, respectively. * means *p* < 0.05, and ** means *p* < 0.01.

The effect of the interleukins on the expression of CD44 and ABCG2 in LNCaP and PC-3 cells was examined by flowcytometry. Typical photos of the flowcytometry and corresponding histograms are shown on Figure 7A. Compared with the control groups, IL-3, IL-6 and IL-11 application in LNCaP cells significantly increased the expression of CD44 and ABCG2 with *p*-values of 0.042, 0.037 and 0.015 for CD44 expression and 0.001, < 0.001, and 0.001 for ABCG2, respectively, while the IL-10 and IL-24 application significantly decreased the expression of CD 44 and ABCG2 with *p*-values of 0.003 and 0.015 for CD44, and 0.010 and 0.027 for ABCG2 as shown on Figure 7B, 7C and 7D. Similar results for PC-3 cells were obtained. Compared to the control PC-3 cells, IL-3, IL-6 and IL-11 application in PC-3 cells significantly activated the expression of CD44 and ABCG2 with *p*-values of 0.017, 0.004 and 0.011 for CD44, and 0.001, 0.006 and 0.008 for ABCG2, respectively. Again, the application of IL-10 and IL-24 resulted in significantly lower levels of CD44 and ABCG2 expression in PC-3 cells, with *p*-values of 0.002 and 0.021 for CD44 and 0.004 and 0.009 for ABCG2 as shown on Figure 7E, 7F and 7G, respectively.

## DISCUSSION

It is known the ILs, a subset of cytokines, actively influence molecule signaling and cellular behavior [41], such as cell proliferation, migration, invasion and adhesion. Increasing evidence points to the important roles of ILs in the microenvironment of tumors. Tumors may rapidly progress when the microenvironment is favorable, and the tumor cells may develop dormancy when the microenvironment allows for such a status. Tumor cell dormancy and tumor cell micrometastasis are two major issues for cancer treatment failure. Although it is known that increasing tumor cell stemness is associated with the features of tumor cell dormancy and micrometastases, molecular mechanisms are still obsolete.

In our current study, the effect of ILs on cell proliferation was examined by a SRB assay, and the results show that IL-3, IL-6 and IL-11 all have similar role in stimulating cell growth in the LNCaP and PC-3 cells. Wound healing and transwell assays also show that these ILs promote migration and invasion of these cells. The cells treated with these ILs show significantly higher colony formation ability and lower number of apoptotic cells, an indication of higher cell stemness in these cells after ILs treatment. In deed, these cells show increasing expression of stemness factors SOX2, and CD44, in addition to the drug-resistant gene ABCG2.

Interestingly, it is already reported that IL-3 is one of the primary factors capable of supporting the growth and acting on early progenitors [42]. Injection of IL-3 in the peripheral blood can maintain more CD34(+) /CD36(+) double-positive erythroid progenitors, which can significantly mobilize the peripheral blood (MPB) CD34(+) cells and increase the repopulating cells in marrow [43]. Presence of IL-3 in cell cultures can support the mesenchymal stem cells expansion of the irradiated CD34(+) cells *in vitro* [44]. It has been suggested to combine IL-3 and G-CSF injection in peripheral blood for peripheral blood stem cell propagation and allogenic stem cell transplantation [45, 46]. Recently, IL-3 hypersecretion has been reported to be associated with cutaneous B-lymphoblastic lymphoma [47]. Our study reveals another aspect of IL-3 functions, namely stimulating the stemness of prostate cancer cells.

IL-6 has been shown to be a major contributing factor in growth and progression of ovarian cancer [48], celiac disease [49] and neck squamous cell carcinoma [50]. It has been reported that the early growth response 3(EGR3) directly activates the excessive production of IL-6 in prostate cancer and promoted the progression [51]. Autophagy, a critical process for breast cancer stem cells (CSC), maintains the cell stemness of breast CSC by regulating the secretion of IL-6 [52]. Chang and co-workers [53] reported that IL-6 induces the expression of OCT4/NANOG and then activates the IGFIR to promote the progression of HBV [53]. Several reports show that IL-11 stimulate and accelerate the development of ulcerative colitis[54], promote the tumor progression by activating the STAT3 and suppress the antitumor immune response [55]. Furthermore, higher levels of IL-11 expression have been reported in distant metastatic gastric cancer cells [56]. The results are in line with our present study showing that IL-6 and IL-11 stimulated the stemness of prostate cancer cells *in vitro*.

On the contrary to the above findings for ILs-3, 6 and 11, we have demonstrated that IL-10 and IL-24 inhibit the proliferation capability of LNCaP and PC-3 cells. All other experiments for migration and invasion, colony formation, chemotherapeutic effect and the expression of stemness factors etc. show opposite results than using ILs-3, 6, and 11, indicating that these two factors suppressed the stemness of these cancer cells *in vitro*.

Contradictory observations for the role of IL-10 in cancer have been reported. Beguelin and associates [57] reported that IL-10 promotes tumor cell proliferation and survival by STAT3 signal pathway, and blocking of IL-10 receptor was suggested as a novel therapeutic target in diffused large B-cell lymphoma [57]. Others have reported that absence of IL-10 to increase the risk of acute lymphoblastic leukaemia occurrence (ALL) [58], and the polymorphisms of IL-10 may be associated with an increasing risk of colorectal cancer [59]. Nevertheless, accumulating evidence indicates that IL-10 is involved in the prostate cancer progression [60, 61], as well as breast [62] and non-small cell lung cancers [63]. We have shown that IL-10 significantly inhibit the growth, migration and invasion, most probably by down regulating the cell stemness in these two prostate cancer cell lines *in vitro*.

IL-24 is a member of the IL-10 family [41]. Increasing number of studies has pointed out the possibility of IL-24 as a promising therapeutic target for tumors. IL-24 inhibits the growth of breast [64] and lung [65]cancers. It is also reported that IL-24 induces apoptosis in melanoma cells [66], and enhances antitumor activities when applied in combination with paclitaxel in breast [67] and prostate [68] cancers. Recombinant human IL-24 (rhIL-24) reverses the chemoresistance in human breast cancer cell line MCF-7 cells [69] and significantly suppresses the growth of ovarian cancer cell [70]. It is also reported that IL-24 plays a critical antitumor role in oral [71], rectal [72] and pancreatic cancers [73]. To our best knowledge, this is the first report exploring the effect of IL-24 on the stemness of prostate cancer cells *in vitro*.

In summary, we have discovered in our present study that while ILs-3, 6 and 11 have similar tumor promotion and stemness stimulation effects, IL-10 and IL-24 reveal opposite effects on prostate cancer cells *in vitro*, underlining a complex role of ILs *in vivo*, which merit further studies.

## MATERIALS AND METHODS

### Cell culture

Human PCa cell lines LNCaP and PC3 cells were obtained from the Chinese Academy of Sciences (ATCC, USA). All cells were cultivated in RPMI 1640 (Gibco/Invitrogen, USA) medium supplemented with 10% fetal bovine serum (FBS) (Hyclone, USA), 1% penicillin/streptomycin (Sigma-Aldrich, USA) and L-glutamine (Sigma-Aldrich, USA) in a humidified 5% CO2 incubator at 37°C.

### Sulforhodamine B assay

Human PCa LNCaP and PC3 cells were plated at 6,000 per well in 96-well plates and incubated overnight at 37°C in a humidified incubator containing 5% CO2. On the following day, IL-3, 6, 10 and 11(Invitrogen, USA) and IL-24(R&D, USA) up to 100 ng/ml (0.01, 0.1, 1, 2,5,10, 50, 100 ng/ml) in complete medium were added to different wells and cultivated for additional 48 hrs. Control wells were added with complete medium. Cell viability was determined using the Sulforhodamine B assay (SRB) (Sigma, USA) according to the manufacturer's instructions. Briefly, culture medium was aspirated and the cells were fixed by addition of 100 μl cold 10% trichloroacetic acid (TCA) at 4°C for 1 h, washed five times with deionized water and left to dry at room temperature. The cells were then stained with 100 μl SRB dye 0.4% (w/v) dissolved in 1% acetic acid (v/v) for at least 15 minutes, washed four times with 1% acetic acid to remove unbound dye and left to dry at room temperature. The dye bound protein was solubilized with 150 μl 10 mM unbuffered tris base and examined with Multi-Mode Microplate Reader (Biotek Synergy2, USA) for optical density reading at 560nm.

### 5-ethynyl-2′-deoxyuridine assay

Cells were plated at 5 × 10^3^ per well in 96-well microtiter plates, treated with different cytokines and incubated for 24 hrs. Then 100 μl 50 μM 5-ethynyl-2′-deoxyuridine (EdU) (CellLight EdU DNA imaging Kit, Guangzhou RiboBio, China) were added into each well and the cells were cultured for an additional 2 hrs. The cells were stained with EdU according to the manufacturer's protocol. EdU medium was discarded and 4% paraformaldehyde was added to fix the cells at room temperature for 30 min. The cells were washed with glycine (2 mg/ml) for 5 min in a shaker and treated with 0.5% Trion X-100 for 10 min before washed with PBS for five minutes. The cells were then incubated with 1× Apollo® click stain reaction buffer for 30 min while protecting from light, washed with 0.5% Triton X-100 for three times to permeate the cells, stained with Hoechst33342 (10ug/ml) for 30 min at room temperature, washed with PBS for three times. The cells were examined with an inverted florescence microscope(Olympus, Japan) immediately.

### *In Vitro* wound healing assay

Cells were seeded into a 6-well plate and allowed to grow to 60% confluent in complete medium. Cells were then wounded by a sterile pipette tip (1 mm), washed with PBS for several times to remove cell debris and incubated for additional 18 h with serum-free RPMI 1640 medium with or without corresponding interleukins. During the incubation at 37°C, cells migrated into wound surface which was considered as a process of *in vitro* healing. The healing process was recorded by inverted fluorescence microscopy. The rate of wound healing=[(the wound width of 0 h- the wound width 18 h)/the wound width 0 h wound width] × 100%.

### Transwell migration and invasion assay

Transwell 24-well filters (Corning, USA) with 8.0 μM pores were used for the migration and invasion assays, according to the protocols recommended by the manufacturer. Briefly, for invasion assay, transwell membranes were coated with 60 μL of matrigel (BD, USA) at a final concentration of 0.1 mg/mL and dried 1 × 10^5^ cells in 100 μL with serum-free RPMI 1640 medium. The membranes were added to the upper chamber triplicate wells and allowed to migrate through matrigel overnight at 37°C with 5% CO_2_ in a humidified incubator. The lower compartment of the transwell chamber was filled with 600 μL RPMI 1640 with 10% FBS. The migration assay was performed with exactly the same procedure except that the membranes were not coated with matrigel. After incubated for 24 hrs(migration assay was 6 h), the cells on the upper surface of filter were removed with a cotton swab, fixed with 4% formaldehyde and stained with Giemsa solution. The cells on the lower surface, which were the migrated/invaded cells, were photographed under the high-power microscopic field(HPF)(200 ×). The rate of migration/invasion inhibition = [(OD values of the control group - OD values of the transfection group)/OD values of the control group] × 100%.

### Flow cytometry analysis

All cells were harvested at logarithmic growth phase before analyzed by a flow cytometer (FCM, FACSCalibur, BD, USA) within 1 h.

Apoptotic cells were detected using Annexin V-FITC/PI kit(KeyGen Biotech, China) according to manufacturer's instructions. Briefly, after treated with interleukin that combined with and without docetaxel(10nmol/L), cells were centrifuged at 1000 rpm for 5 min at the concentration of 1 × 10^6^ cells/ml. Then the pellets were washed twice with ice-cold phosphate buffered saline (PBS) and resuspended in 500 μl binding buffer before 10 μl Annexin V-FITC and 5 μl of PI were added into each of the solution, and the cells were gently vortexed and incubated for 15 min at room temperature in the dark before flowcytometry.

For cell surface stem cell markers ABCG2 and CD44, an anti-ABCG2 monoclonal antibody directly conjugated with phycoerythrin(PE) and an anti-CD44 monoclonal antibody directly conjugated with APC, purchased from BD Pharmingen Company, were applied in this study. The antibodies were used at optimized dilutions, and the cells were prepared with a similar procedure as described above before flowcytometry. PE Mouse IgG2b (eBioscience, USA) and APC Mouse IgG2b (eBioscience, USA) isotype controls were used as negative controls. Viable and single cells were gated for each sample before examination

### FQ-RT-PCR

Total RNA was extracted using TRIzol reagent (Invitrogen, USA) by following the manufacturer's instructions. Briefly, after cells were treated with or without IL-3, 6, 10, 11 and 24 at 48 hrs, the cells (1 × 10^6^) were harvested and washed twice with cold phosphate-buffered saline (PBS). For each well, 1.0 mL TRIzol reagent was added, and then RNA was precipitated by isopropanol, washed with 75% ethanol, dissolved with 20 μL DEPC (0.1%), and quantified using a UV spectrophotometer.

Reverse transcription was carried out with the PrimeScript™RT reagent Kit (TaKaRa, Janpan) according to the standard protocol. In brief, in a 20 μL reaction mixture containing 2 μg RNA and oligo primers, at 42°C for 1 h, and the synthesized cDNA was used for PCR by using SYBR®Premix Ex Taq™II (TaKaRa, Dalian, China) with the primers as shown in Table 1. PCR was performed as follows: 30 sec of pre-degeneration at 95°C; 5 sec at 95°C and 20 sec at 60°C for 40 cycles with Roche instrument- Lightcycler 480 (Roche, USA). SOX2 gene and β-actin gene were amplified in the same reaction where β-actin gene was applied as an internal loading control. The amplification specificity was confirmed by the melting curves, and the fluorescence was collected at 60°C (*n* = 3), and the relative quantitative results were analyzed by the 2^−ΔΔCt^ values.

**Table 1:** Sequences of the primers used for RT-PCR

Gene	Accession No.	Sequence(5′to3′)
SOX2	NM_003106	F- GTGAGCGCCCTGCAGTACAA R- GCGAGTAGGACATGCTGTAGGTG
β-actin	NM_017008	F- CAAGG TCATCCATGACAACTTTG R- GTCCACCACCCTGTTGCTGTAG

### Western blotting

Cells ready for Western blotting analysis were harvested and washed with cold PBS twice, then lysed on ice in RIPA buffer (1 × PBS, 1% NP-40, 0.1% sodium dodecylsulfate (SDS), 5 mM EDTA, 0.5% sodium deoxycholate, and 1 mM sodium orthovanadate) that contained 100 μg/mL phenylmethylsul-fonylsuoride and protease inhibitors (KeyGen, Nanjing, China).

Approximately 50 μg of protein from each sample was separated using a 10% SDS-polyacrylamide gel, electrotransfered to polyvinylidene fluoride(PVDF) membranes and blocked in 5% nonfat dry milk in Tris-buffered saline, pH 7.5 (100 mM NaCl, 50 mM Tris, and 0.1% Tween-20). The transferred membranes were incubated with anti-SOX2 (Cell Signaling Technology, USA) and anti-β-actin primary antibodies (Beyotime, Jiangsu, China) overnight at 4°C, followed by incubation with horseradish peroxidase(HRP) conjugated IgG(JacksonImmunoResearch, USA). Proteins were detected by Quantity-one software (Bio-Rad, Laboratories, Inc, USA) using Immobilon ECL Chemiluminescence HRP Substrate (Millipore, Merck, USA).

### Immunofluorescence microscopy

2 × 10^5^ cells were plated on coverslips, which were placed on the bottom in 6-well plates, and treated with different cytokines. Before examination, the cells were fixed with 4% paraformaldehyde in PBS(pH 7.5) for 30 min and permeated with 0.5% Trion X-100 for 15 min at room temperature. The coverslips were first immersed for 1 h in blocking solution that contained 5% bovine serum albumin (BSA) in PBS, and the cells were then incubated for overnight at 4°C with rabbit antibodies against SOX2 (CST, USA). DNA was counterstained with 4′,6-diamidino-2-phenylindole (DAPI, 5ug/ml) and observed under inverted fluorescence microscope.

### Colony formation assay

Single LNCaP and PC3 cells (200 cells/well) were planted in 35 mm well plates for overnight incubation to allow for cells attachment. Different interleukins were then added into the medium for culturing another two weeks. The cells were then fixed with 4% paraformaldehyde for 30 min, and stained with Giemsa for 30 min at room temperature. The plates were gently washed with PBS and evaluated under microscope. Cell cluster with more than 30 cells was considered as a colony. Colony formation efficiency was estimated as follows: Colony formation efficiency = colonies/input cells × 100%.

### Statistical analysis

All data are expressed as mean ± the standard deviation (SD) and subjected to one-way analysis of variance (ANOVA). Differences between groups were examined by Student t test unless otherwise noted. Statistical significance was accepted at the level of *p*-value less than 0.05 by using SPSS 17.0 software.
